# The Role of MAPK in Drug-Induced Kidney Injury

**DOI:** 10.1155/2012/463617

**Published:** 2012-03-12

**Authors:** Hilary Cassidy, Robert Radford, Jennifer Slyne, Sein O'Connell, Craig Slattery, Michael P. Ryan, Tara McMorrow

**Affiliations:** Renal Disease Research Group, School of Biomolecular and Biomedical Sciences, UCD Conway Institute, University College Dublin, Belfield, Dublin 4, Ireland

## Abstract

This paper focuses on the role that mitogen-activated protein kinases (MAPKs) play in drug-induced kidney injury. The MAPKs, of which there are four major classes (ERK, p38, JNK, and ERK5/BMK), are signalling cascades which have been found to be broadly conserved across a wide variety of organisms. MAPKs allow effective transmission of information from the cell surface to the cytosolic or nuclear compartments. Cross talk between the MAPKs themselves and with other signalling pathways allows the cell to modulate responses to a wide variety of external stimuli. The MAPKs have been shown to play key roles in both mediating and ameliorating cellular responses to stress including xenobiotic-induced toxicity. Therefore, this paper will discuss the specific role of the MAPKs in the kidney in response to injury by a variety of xenobiotics and the potential for therapeutic intervention at the level of MAPK signalling across different types of kidney disease.

## 1. Introduction

Intracellular signalling cascades are the primary routes of communication between the plasma membrane and regulatory targets in various intracellular compartments. Sequential activation of kinases is a widely conserved mechanism of signal transduction in many cellular processes. Protein kinases are ubiquitous enzymes that modulate the activities of other proteins through the addition of phosphate groups to tyrosine, serine, or threonine amino acid residues, a process referred to as phosphorylation. Over the last decade, several related intracellular signalling cascades, collectively known as mitogen-activated protein kinase (MAPK) signalling cascades, have been characterized. MAPKs belong to a large group of serine/threonine protein kinases which have been shown to be conserved in organisms as diverse as yeast and humans [1–3], and which can be activated by numerous extracellular stimuli [4, 5]. In combination with several other signalling pathways, MAPKs can differentially alter the phosphorylation status of numerous proteins including transcription factors, cytoskeletal proteins, kinases, and other enzymes and can significantly influence gene expression, metabolism, cell division, cell morphology, and cell survival. Epigenetic aberrations of these enzymes, or of the signalling cascades that regulate them, have been implicated in a variety of human diseases including cancer, inflammation, and cardiovascular disease. Dysregulation of normal MAPK signalling has also been implicated in both acute and chronic kidney disease. In this paper we focus on the role of MAPKs in kidney disease, and in particular, the role that MAPK signalling plays in drug-induced kidney injury and disease.

## 2. The MAPK Family in Kidney Injury

**Figure 1:**
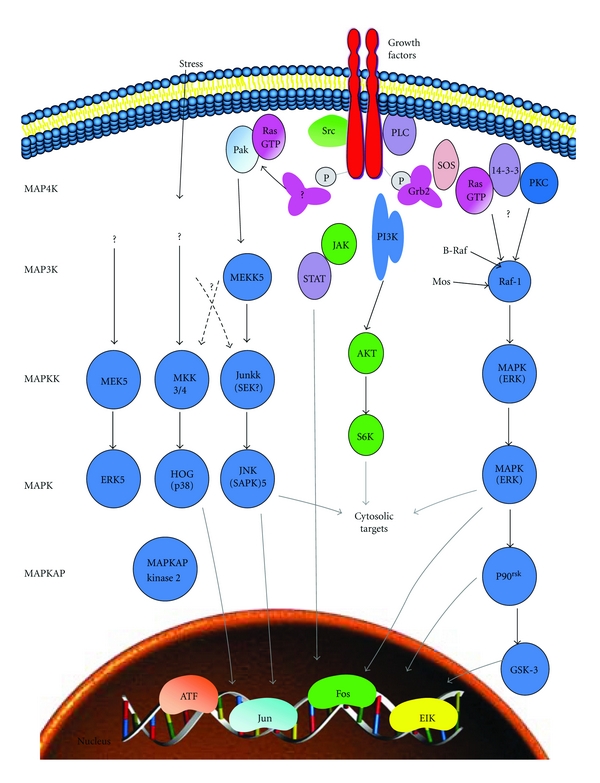
The MAP kinase signalling pathways (adapted from http://www.sabiosciences.com/).

The transmission of extracellular signals to various intracellular targets is a multifaceted process which often involves the activity of one or more MAPKs. The process begins in response to external stimuli such as binding of a growth factor to its associated receptor on the cell surface. The resulting activation of the integral or associated protein tyrosine kinases contained within the intracellular domain of the receptor then initiates intracellular signalling events. Activation of a MAPK employs a core three-kinase cascade whereby a MAPK kinase kinase (MAP3K or MAPKKK) phosphorylates and activates a MAPK kinase (MAP2K or MKK) which in turn phosphorylates one or more MAPKs [6–8]. An overview of the signalling pathways is outlined in Figure 1. Once activated, MAPKs can phosphorylate several different intracellular targets including transcription factors, nuclear pore proteins, membrane transporters, cytoskeletal elements, and other protein kinases [8–10]. This three-tier module mediates ultrasensitive switch-like responses to stimuli. Uniquely, MAPKs themselves can be activated by addition of phosphate groups to both their tyrosine and threonine amino acids (dual phosphorylation) after stimulation of a receptor by growth factors, mitogens, hormones, cytokines, or environmental stresses [11]. These various members of the MAPK family have been duplicated with slight variations, allowing cells to instigate multiple biological responses through a set of MAP kinase networks.

### 2.1. ERK 1/2 Signalling Cascade

**Figure 2:**
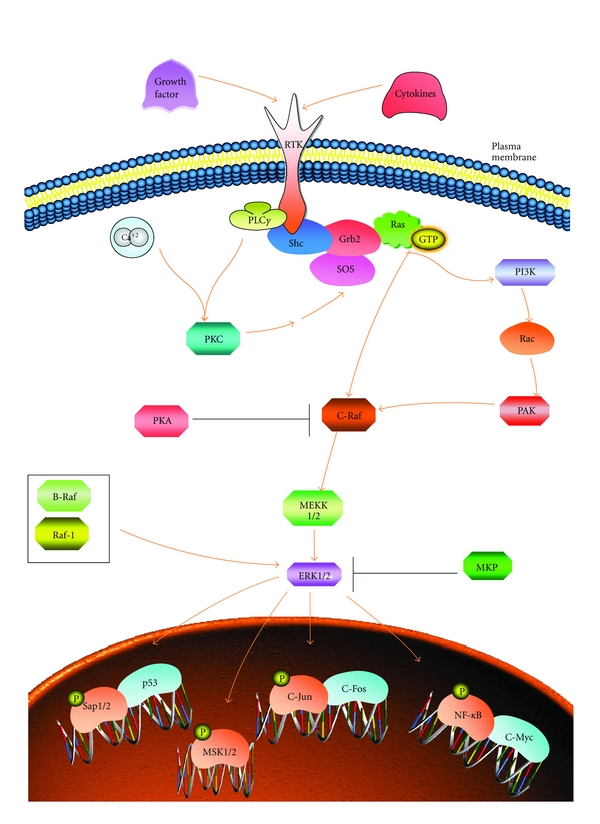
Structure of the ERK pathway (adapted from http://www.sabiosciences.com/). Upon ligand binding, RTK autophosphorylates on tyrosine residues, which serve as docking sites for adaptor and signalling molecules. Ras is activated by the recruitment of guanosine-nucleotide exchange factors (SOS, C3G) via adaptor proteins (Shc and Grb2; Crk). Ras can activate Raf-1 and B-Raf; Rap1 presumably can activate B-Raf. Raf proteins phosphorylate and activate MEK-1/2, which in turn activate ERK-1/2 (indicated by black arrows).

In the early 1980s, the 42 kDa extracellular signal-regulated protein kinases 1 and 2 (ERK1/2) were the first members of the MAPK family to be identified and cloned from a vertebrate species [12–16]. ERK1/2 functions have been linked to the regulation of growth and differentiation [17, 18]. ERK1/2 activation is mediated by the specific protein kinases MAPK/ERK kinases (MEK) 1/2, which are members of the MAPK supergene family MEK2. MEK1 and MEK2 are encoded by different genes, but are very similar in terms of sequence, substrate specificity, and regulation [19, 20]. MEK1/2 themselves are activated through phosphorylation by three distinct MAPKK kinases (MAP3K or MAPKKK), Raf, c-Mos, and MEK kinase (MEKK). The major pathway by which tyrosine kinase mediated signals are directly conveyed to the ERK1/2 cascade is through Ras-mediated recruitment of Raf to the plasma membrane. Ras activation is dependent on the tyrosine-kinase-mediated membrane translocation of the guanine nucleotide exchange factor SOS by the Grb2 adapter protein, which connects SOS to the tyrosine-phosphorylated receptor [21]. Elk-1 and SRF are target genes regulated by ERK1/2 [6]. An overview of the ERK1/2 signalling pathway is outlined in Figure 2.

ERK signalling plays a major role in mediating renal cell responses to a diverse range of stimuli and has previously been shown to be involved in compensatory renal hypertrophy and pathological conditions such as glomerular and tubulointerstitial diseases. Robust ERK activation has been detected in the cyst epithelium in polycystic kidney disease (PKD), while inhibition of the ERK pathway led to inhibition of cyst-induced gain in kidney mass and improved renal function [22]. In instances of drug-induced nephropathy such as kidney injury associated with cisplatin administration, stimulation of ERK occurs via the EGF-R/Src cascade [23]. Cisplatin-induced nephrotoxicity is dependent on DNA damage-induced apoptosis. Conversely, inhibiting ERK *in vivo,* in a rat model of progressive membranous nephropathy (PHN), was demonstrated to worsen DNA damage observed in the podocytes and resulted in an upregulation of p21, suggesting a protective role of ERK in this model [24]. This suggests a dual nature of this particular MAPK: on the one hand mediating a kidney injury response while on the other hand playing a role in renal defence.

### 2.2. JNK Signalling Cascade

**Figure 3:**
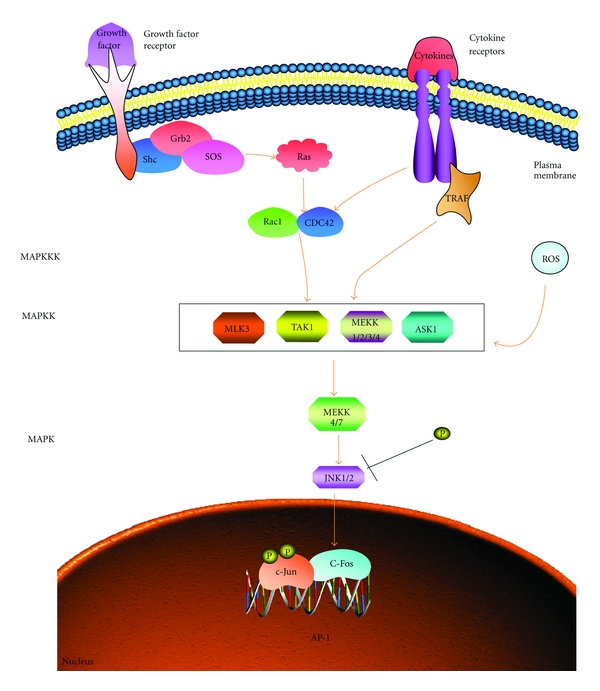
The JNK signalling pathway (adapted from http://www.sabiosciences.com/). Upstream events are poorly defined, but in the case of growth factors and the cytokine TNF are known to involve G proteins such as Rac and cdc-42 and the protein TRAF2. Activated JNK1 and JNK2 isoforms phosphorylate the AP-1 subunit c-Jun, increasing its transcriptional activity. ASK1, apoptosis signal-stimulating kinase 1; MLK, mixed-lineage protein kinases; TAK1, transforming growth factor-*β*-activated kinase-1.

The JNK cascade was first discovered through studies on the oncogenic cooperation between Ras and the target JNK transcription factor c-Jun, and on the activation of transcription factor AP-1 by UV irradiation [25]. Both studies indicated that phosphorylation of a component of AP-1 at Ser63 and Ser64 occurred in response to either Ras activation or UV irradiation such phosphorylation, especially at Ser73, enhances the ability of c-Jun to activate gene transcription [26]. The protein kinases that bind to c-Jun and phosphorylate it at Ser63 and Ser73 were subsequently identified as JNKs. There are two JNK polypeptides, the products of two distinct genes, and they share 81% sequence identity [27]. Like all MAPKs, the JNKs are activated by phosphorylation on conserved threonine and tyrosine residues. Consistent with the different sequences surrounding their activating phosphorylation sites, the JNKs are not phosphorylated by MEK1/2 but by another MAPKK, named SEK 1, MKK4 or JNKK1. Similar to other MAPKKs, JNKK1 is phosphorylated and activated by a MAP3K called MEKK1 [28]. An overview of the JNK signalling pathway is outlined in Figure 3. Activation of JNKK1 by MEKK1 was demonstrated *in vitro *[29]. Very strong JNK activation is observed after irradiation of cells with either UV light or treatment with certain translational inhibitors such as anisomycin, exposure to IL-1, costimulatory activation of T-cells, ischemia reperfusion, and exposure to alkylating agents. JNK activation is also observed after treatment of certain cell types with growth factors such as EGF and NGF. The mechanisms by which these stimuli activate the JNK cascade have not been elucidated. The majority of experimental analysis has focused on the mechanisms of JNK activation by growth factors and members of the Src family of tyrosine kinases [28, 30].

In the context of renal injury, JNK signalling is mediated by different insults including ischaemia/reperfusion (I/R), ureteric ligation, immune-mediated injury, and hyperglycaemia [31, 32]. JNK activation has been demonstrated in several glomerulonephritides [33], and JNK inhibition suppresses inflammation in rat antiglomerular basement membrane disease [34] and also suppresses tubular apoptosis and interstitial fibrosis in unilateral ureteral obstruction (UUO) models [35]. Kanellis et al. demonstrated that acute activation of JNK signalling occurs following I/R, with higher JNK activation detected in deceased donor compared to live donor allografts, thus suggesting that increased JNK reflects greater ischaemic damage [36]. Administration of a JNK inhibitor prior to I/R injury prevented tubular damage and renal dysfunction, suggesting involvement of JNK activation in both cellular rejection and acute tubular necrosis [36].

### 2.3. P38 Signalling Cascade

**Figure 4:**
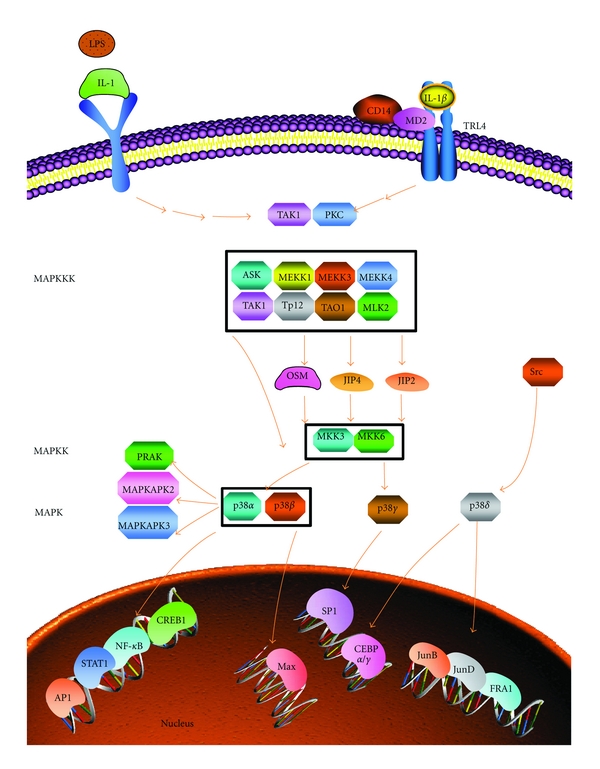
The p38 signalling cascade (adapted from http://www.sabiosciences.com/). RNA-binding proteins mediate regulation of cytokine mRNA stability through p38/MK2 signalling. LPS activates p38 MAPK signalling in a variety of cells, leading to transcriptional activation of cytokine genes or enhanced mRNA stability and translation (highlighted areas). The fate of adenine- and uridine- (AU-) rich elements (ARE) mRNA is dependent on the presence of destabilizing and stabilizing mRNA-binding proteins. p38 MAPK activates MK2 in the nucleus, allowing for MK2 translocation to the cytoplasm. MK2 subsequently phosphorylates destabilizing mRNA-binding proteins such as TTP. This action prevents TTP from interacting with ARE cytokines. Simultaneously, activation of the p38 MAPK pathway results in translocation of HuR, a stabilizing RNA-binding protein, from the nucleus to the cytoplasm. Thus, upon p38/MK2 activation and phosphorylation of TTP, cytokine mRNA stability is enhanced, because TTP is no longer dictating mRNA triage and exonuclease decay.

p38 is another MAPK protein, which is most similar to the yeast MAPK HOG-1 which is activated in response to osmotic shock [37]. Like HOG-1, p38 is also activated in response to osmotic shock, as well as by LPS and IL-1. For the most part, p38 also responds to the same agonists that activate the JNKs. The similarity between the regulation of p38 and JNK is not surprising since JNKK1 (SEK1) is also a direct activator of p38. Downstream targets of p38 include the genes MAPKAP kinases 1 and 2 and ATF-2 [38, 39]. p38 MAPK is an important regulator of senescence growth arrest due to its ability to activate both the p53 and pRb/p16 growth arrest pathways. p38 MAPK inhibition has been demonstrated to moderately delay replicative senescence [40]. Furthermore, p38 MAPK activity is required for the senescence arrest caused by oncogenic RAS, and constitutive p38 MAPK activity can induce a growth arrest in normal human cells [41, 42]. p38 MAPK is known to upregulate specific cytokines such as IL-6, IL-8, and TNF*α* in several biological contexts including kidney damage [39, 43, 44]. An overview of the p38 MAPK signalling pathway is outlined in Figure 4. p38 MAPK activity has been shown to be necessary and sufficient for the development of a senescence-associated secretory phenotype (SASP). SASP is a persistent, nonacute inflammatory response in cells which have been induced to senescence by direct DNA damage or oncogenic RAS [45–47].

It has been reported that inhibition of p38 MAPK in autoimmune renal disease reduced the severity of the disease, resulting in a prolonged life span in MRL-*Fas^lpr^* mice [48]. This protection against renal injury *in vivo *resulted from reduced infiltration of leukocytes, diminished expression of cytokines which are known to promote renal injury, and reduced production of Ig, leading to the conclusion that activation of p38 MAPK is required to promote cytokine/chemokine and Ig production, which in turn result in lethal autoimmune renal injury *in vivo*. Stambe et al. localized components of the p38 MAPK pathway to podocyte-like cells, endothelial cells, and infiltrating neutrophils in a model of acute renal inflammation [49]. A similar pattern of p38 activation was observed in postinfectious glomerulonephritis [49]. It was shown that blockade of the p38 pathway significantly inhibited acute renal failure and proteinuria in rat anti-GBM glomerulonephritis via a neutrophil-platelet and P-selectin-dependent mechanism, thus suggesting that blockade of the p38 MAPK pathway may be a novel therapeutic strategy for the treatment of acute renal inflammation.

### 2.4. Ras Signalling

Ras is a small GTP-binding protein with multiple effector molecules, each of which defines a pathway with specific functions. Ras has been implicated, to different extents, as a mediator of ERK, JNK, and p38 activities. p38 has been implicated in cell motility stimulated by PDGF, in a Ras-dependent pathway [50]. Phosphatidylinositol 3-kinases (PI3K) have been suggested to be involved in cell motility through Ras-mediated activation of Rac. Rac is a member of the Ras superfamily of small GTP proteins which has a well-established role in cell migration and invasiveness [51–53]. Rac can be activated by Ras directly via recruitment to the cell membrane or via Ras activation of PI3Kinase. In contrast to its functions in motility, Rac has been shown unambiguously to strengthen cell-cell adhesion thereby preventing tumour cell invasiveness [54, 55]. Studies have shown these opposing effects of Rac arise due to dependency on the cell substrate [56]. On substrates permissive for locomotion, expression of active Rac promotes motile behaviour, whereas, on substrates impeding cell motility, Rac-dependent cell-cell adhesion is favoured.

Other members of the Ras superfamily of small G proteins are Rho and Cdc42. Along with Rac, Rho and Cdc42 control different aspects of the cytoskeleton and seem to act in a cascade in which Cdc42 acts upstream of Rac which in turn acts upstream of Rho. Rho displays complex functions in cell scattering and it is involved in the assembly of focal contacts and actin stress fibres in fibroblast cells [57, 58]. Rho plays a positive role in colony-stimulating factor-1 induced macrophage translocation and in the migration and metastatic properties of human hepatocellular carcinomas [59]. Rho carries out these actions by stimulating the phosphorylation of myosin light chain and adducin, an actin-binding protein [60]. Like Rac, Rho can also have antagonistic effects on epithelial cell scattering by reinforcing cell-cell adhesion sites. Again, like Rac, the contrasting Rho activity is cell substrate dependent [61].

Activated Ras is sufficient for full ERK activation but is only a weak JNK activator producing about one-third of the JNK activity observed after treatment with EGF or expression of v-Src. Rac and cdc42 are effective JNK activators and can act synergistically with Ras. It is proposed that Rac in turn can stimulate Pak1 via cdc42, which could induce phosphorylation of myosin light chain, thus linking this Ras/Rac-induced pathway to proteins directly affecting cell movement [37, 62]. Pak1 has been demonstrated to induce modest activation of JNK suggesting autocrine upregulation of JNK signalling.

### 2.5. Src Signalling

Src tyrosine kinase is another positive regulator of growth-factor-induced cell scattering [63]. Src belongs to a family of cytoplasmic tyrosine kinases. These enzymes have a pivotal role in the regulation of a variety of biological functions which are associated with changes in morphology, including malignant transformation [64, 65], epithelial plasticity [66], and modulation of intercellular adhesion [67]. In addition, the Src family is required during mitogenesis induced by EGF, PDGF, and colony-stimulating factor-1 [68, 69]. Src regulates much of the activities of the JNK and p38 pathways. There are nine members in the Src family; Src, Fyn, and Yes are ubiquitously expressed while the other members have a more restricted pattern of expression. There is a possibility of redundancy of function amongst the Src family members [70] so the specificity of action of each individual member is not clear. This redundancy can arise by the phosphorylation of common substrates important for signalling, and it is probable that Src and Yes have redundant functions during cell scattering [71]. Three mechanisms by which Src kinases exert their functions have been suggested. Firstly, Src may phosphorylate specific substrates that are mainly cytoskeletal-based components, and molecules localised in cell-cell and cell-substrate adhesion sites, tyrosine phosphorylation of which could in turn alter cellular architecture [72–75]. Secondly, by inducing Myc via a specific transduction pathway, Src may also participate in entry into S-phase [76]. Finally, Src could interact with other signalling pathways, as it has been shown that it is capable of binding Shc, an early element of the Ras cascade leading to the activation of this pathway [77, 78].

A role for Src signalling in the repair mechanisms of acute kidney injury has also been indicated. A recent study by Takikita-Suzuki et al. has provided evidence to suggest the importance of active Src kinase in the early phase of PDGF-B-dependent nephrogenic repair after acute ischemia/reperfusion injury and has identified the distribution of active Src kinase in the normal and reperfused kidney [79].

## 3. Drug-Induced Kidney Injury

The anatomical, biochemical, and physiological specialisations which permit the kidney to perform its vital roles in homeostasis may increase the risk of drug exposure to the components of the nephron, and its ancillary structures. The kidneys receive 25% of cardiac output, filtering 180 L of plasma, producing 1.5 L of urine each day. As a result, high levels of drugs may be concentrated, leaving the epithelium of the nephron at a significantly greater risk of damage from pharmaceutical agents. In addition, the kidney is a metabolically active organ which contributes significantly to metabolism of xenobiotics. While renal metabolism usually contributes to the detoxification of xenobiotics, there are instances whereby substances undergo bioactivation to more toxic metabolites [80]. In addition to glomerular filtration, removal of xenobiotics and waste products from the blood can occur via transcellular transport, which transports compounds directly from the blood into the lumen by organic ion transporters. This organic ion transport system may allow toxic compounds to accumulate in the cells of the nephron which may otherwise not gain access to the cytosol [81].

## 4. Antibiotic-Induced Kidney Injury

### 4.1. Antibiotics

Antibiotics are a group of drugs used to treat various infections caused by bacteria and other microorganisms. The first antibiotic was discovered by Sir Alexander Fleming in 1928 in a significant breakthrough for medical science. The development of antibiotics is probably the largest advance in medicine in the 20th century and has saved millions of lives worldwide from infections such as TB.

Gentamicin is an aminoglycoside broad spectrum Gram-negative antibiotic which is routinely used in clinical practices for the treatment of Gram-negative infections alone or in synergy with beta-lactam antibiotics in adults, children, and neonates. Gentamicin use is however associated with significant nephrotoxicity—it is estimated that roughly 30% of patients receiving gentamicin for 30 days show some signs of renal impairment [82–84]. Gentamicin-induced nephrotoxicity is characterized by morphological alterations including destruction of cell organelles and necrosis, lysosomal swelling and mitochondrial vacuolisation preceding functional alterations marked by proteinuria, increased levels of serum urea and creatinine, which lead to acute kidney injury (AKI). The site of gentamicin nephrotoxic action is the kidney cortex, especially the proximal tubules. Animal models of aminoglycoside nephrotoxicity also present areas of interstitial fibrosis in the renal cortex and progressive tubular injury [85, 86].

Vancomycin is a cationic glycopeptide antibiotic used in the treatment of penicillin-resistant Gram-positive infections, methicillin-resistant *Staphylococcus aureus *infections, and *Clostridium difficile *infections, which has seen a resurgence in use due to the emergence of *β*-lactam-resistant Gram-positive organism-associated infections. Vancomycin is known to be both ototoxic and nephrotoxic [87], with vancomycin-induced nephrotoxicity reported to occur in up to 25% of patients [88, 89]. Studies carried out in animal models showed increased urinary excretion of proximal tubule cells and in the activity of malate dehydrogenase (MDH) following vancomycin administration [90, 91]. Furthermore, a recent study also suggested that oxidative stress might underlie the pathogenesis of vancomycin-induced nephrotoxicity [92].

Acute kidney injury (AKI) is a common side effect of antibiotic therapy. However, given the diversity of the mechanisms of action of the most commonly used antibiotic therapies, it is perhaps not immediately clear whether this injury occurs as a result of a defined process. There is evidence however to implicate involvement of the MAPK signalling cascade in antibiotic-induced renal injury initiated by several distinct classes of antibiotics.

### 4.2. MAPK in Antibiotic-Induced Nephrotoxicity

A study carried out by Volpini et al. in 2006 showed that MAPKs may be involved in the pathogenesis of acute renal failure following gentamicin treatment [93]. Since there is evidence that both the MAPK and NF-*κ*B systems can be activated by oxidative stress in gentamicin-treated animals, the expression of p-p38 MAPK and NF-*κ*B in the kidney during the evolution of tubulointerstitial nephritis and its relationship with histological features and renal function was investigated in gentamicin-treated rats in the presence or absence of an NF-*κ*B inhibitor. Western blot analysis demonstrated the presence of the 43-kDa phosphorylated p38 MAPK and the 65-kDa NF-*κ*B proteins in the renal cortex from all treatment groups compared to control. The gentamicin-treated animals showed a greater p38 expression than the control and NF-*κ*B inhibitor and gentamicin-treated animals. Data obtained in this study showed that p38 MAPK expression is increased during the development of gentamicin-induced interstitial nephritis and that such alteration is associated with enhancement of NF-*κ*B expression and the inflammatory process in the renal cortex, suggesting that the p38 MAPK pathway may be involved in the renal lesions induced by gentamicin [93]. Other studies verify this involvement of MAPK in antibiotic-associated nephrotoxicity; p38-MAPK has been found to be upregulated in rat kidneys following gentamicin treatment, and it has been shown that combination treatment with the lipid-lowering drug, atorvastatin, ameliorated gentamicin-induced nephrotoxicity, through inhibition of p38-MAPK and NF-*κ*B expression [94]. Previous studies have also demonstrated that the proliferative response observed after sublethal toxicant-induced renal injury may be mediated by activation of the MAPK signalling pathway, which is ultimately regulated by bioenergetic capacity [95–98].

A study carried out investigating the effects of vancomycin exposure in renal LLCPK_1_ cells on cell proliferation showed a dose- and time-dependent increase in cell number and total cellular protein [99]. These effects were inhibited by pretreatment with a MAPK inhibitor, PD098059, preventing vancomycin-induced entry into the cell cycle, thus suggesting an association between the cell proliferative effects of vancomycin and the induction of MAPK signalling cascades [99]. 

## 5. Calcineurin-Inhibitor-Induced Kidney Injury

### 5.1. Calcineurin Inhibitors

In 1954, the first successful transplantation of a human kidney was performed between identical twins; the success of which was based on the lack of significant rejection between genetically identical twins, thus circumventing the requirement for immunosuppression [100]. Solid-organ transplantation was not truly implemented until the 1970s following significant technical and pharmacological advances, in particular, the discovery and development of the calcineurin inhibitors (CNIs) [101]. Despite the advances over the past four decades, the majority of renal allografts fail after a period of progressive functional decline which is associated with glomerulosclerosis, tubular atrophy, interstitial fibrosis, and arteriosclerosis, in a process referred to as chronic allograft injury (CAI) or chronic allograft nephropathy (CAN). The deterioration of renal allograft function and structure associated with CAI can occur due to immunological processes (i.e., chronic rejection) and/or a range of simultaneous nonimmunological factors such as CNI-induced nephrotoxicity, hypertension, and infection. The two most commonly employed immunosuppressants are the CNIs cyclosporine A (CsA) and tacrolimus (FK506).

CsA, also known as Sandimmune, is a 1203 dalton, lipophilic, cyclic compound, derived from fungal origins which was discovered in 1976. The first incidence of CsA employed as an immunosuppressant was in 1978, revolutionizing the field of organ transplantation [102]. CsA is primarily renowned as a powerful immunosuppressant for use in organ transplantation to prevent graft rejection in kidney, liver, heart, lung, and combined heart-lung transplants [103].

Tacrolimus, also known as FK506 or Prograf, is a 822 Da, 23-membered macrolide compound (C_44_H_69_NO_12_) that was isolated from a soil microorganism *Streptomyces tsukubaensis* in Japan in 1984, [104, 105]. FK506 is a potent immunosuppressive agent employed worldwide since the early 1990s that is effective in allograft prophylaxis after organ transplantation, for therapy of acute rejection and in treatment of different immune diseases [106, 107]. In 2003, FK506 was used as initial immunosuppression in 67% of kidney recipients and 89% of liver recipients (UNOS United Network for Organ Sharing 2004). FK506 is up to 100 times more potent than CsA [108], which has significantly reduced the incidence and severity of acute rejection rates in organ transplantation [109, 110].

**Figure 5:**
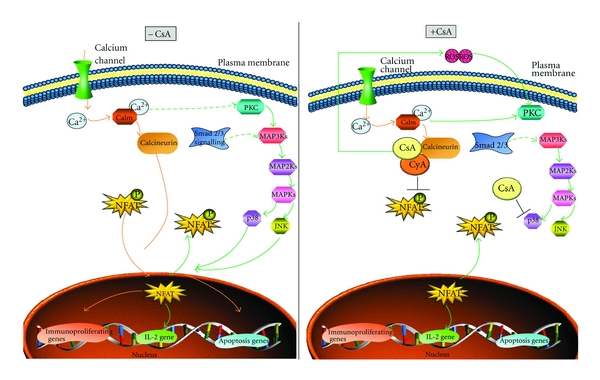
Alternative signaling in the presence of cyclosporine A. In normal, signaling calcineurin binds to and dephosphorylates NFAT, allowing it to enter the nucleus where it induces IL-2 expression. In the presence of cyclosporine this pathway is blocked by the binding of the cyclosporine/cyclophilin complex to calcineurin, which prevents the subsequent binding to NFAT. Alternative signaling occurs via the induction of a MAPK cascade, resulting in the expression of IL-2.

Both CNIs follow similar molecular pathways, with both FK506 and CsA eventually inhibiting NFAT-dependent production of IL-2 and other cytokines and prevention of T-cell growth [111, 112]. Recently, alternative molecular pathways have been identified for CsA, which has been found to inhibit the JNK and p38 signalling pathway activity triggered by antigen recognition in T cells (Figure 5) [113]. 

### 5.2. MAPK in CNI Nephrotoxicity

CsA and FK506 are widely used in transplant organ recipients, but in the kidney allograft, they may cause tubulointerstitial as well as mesangial fibrosis [114]. The fibrogenic effect of CNIs in the renal allograft is predominantly mediated by elevated intrarenal expression of TGF-*β* [57, 115], and subsequent excessive extracellular matrix (ECM) generation [116, 117]. Using the rMC cell line, rat kidney mesangial cells, it has been shown that CsA and FK-506 induce an extremely rapid and dose-dependent increase of Y-Box-binding protein-1 (YB-1) content in a cell type-specific manner. The highly conserved YB-1 is a member of the family of cold-shock proteins with mitogenic properties which play a role in cellular stress responses and tumourigenesis and also controls TGF-*β*1 translation in proximal tubular cells [118–120]. Given the fact that YB-1 is a downstream target of MAPK ERK1/2 [121, 122], the study continued to investigate the involvement of ERK1/2 in CsA-triggered cell activation. Previous studies have documented that YB-1 undergoes phosphorylation at serine 102 (Ser^102^) by activated serine/threonine protein kinase Akt/protein kinase B [114, 123]. Hanssen et al. showed that inhibition of Akt/ERK signals upstream of YB-1 activation prevents its phosphorylation at Ser^102^ and abolishes the CsA-mediated YB-1 protein increase demonstrating that CsA-induced YB-1 accumulation was dependent on MAPK/ERK and PI3K/Akt signalling [124]. Hanssen et al. also verified these results *in vivo*, treating mice either with a vehicle control or CsA and analysing the renal YB-1 content [124]. Immunoblotting indicated elevated YB-1 protein content in the CsA-treated mice, localised in the mesangial compartment [124].

## 6. Cancer-Chemotherapeutic-Agent-Induced Kidney Injury

### 6.1. Cancer Chemotherapeutic Agents

Chemotherapy continues to play a crucial role in the management of cancer, with the basic aim to kill cancerous cells whilst causing minimal damage to the other healthy cells in the body. Cancer chemotherapeutics are divided into different categories with several members in each category, including alkylating agents (e.g., cyclophosphamide); antimetabolites (e.g., methotrexate); plant alkaloids (e.g., etoposide); anthracyclines(e.g., doxirubicin); antitumor antibiotics (e.g., mitomycin C); platinum compounds (e.g., cisplatin); taxanes (e.g., taxol) [125]. Cancer chemotherapeutic agents can cause nephrotoxicity in various ways, with some drugs exerting immediate effects on renal function while others are known to have cumulative effects, causing renal injury after long periods of use [126]. Cisplatin is one of the most successful antineoplastic agents to date. It is used to treat a wide variety of solid tumours, and successful cure rates for certain cancers such as testicular cancer are as high as 90% following cisplatin treatment. One of the major limiting factors in the use of cisplatin however is development of acute kidney injury, with clinically measureable nephrotoxicity usually detected 10 days after administration. It is estimated that 20% of patients receiving high doses of cisplatin develop renal dysfunction [127]. The kidney is particularly susceptible to cisplatin-induced toxicity due to the high concentration of the organic cation transporter 2 (OCT2), which is expressed mainly in the kidney and facilitates cellular entry of the cisplatin compound [128].

### 6.2. MAPK in Cisplatin-Induced Nephrotoxicity

The exact mechanisms governing cisplatin-induced nephrotoxicity are not completely understood; however, it is believed that MAPK plays a pivotal function. Indeed several studies both *in vitro* and *in vivo* have shown that pharmacological inhibition of ERK1/2 ameliorates cisplatin-induced nephrotoxicity. There is conflicting evidence about the role that both p38 and JNK/SAPK play in cisplatin-induced kidney injury. Arany et al. [23] showed that ERK, and not p38 or JNK/SAPK inhibition, prevented cisplatin induced toxicity. However, other studies have shown that pharmacological inhibition of p38 both *in vitro* and *in vivo* prevented toxicity [129–131]. The role that JNK/SAPK plays in acute kidney injury following cisplatin exposure has been less well characterised; however, it has been shown that JNK/SAPK inhibition resulted in a significant reduction in cisplatin-induced nephrotoxicity *in vivo *[132]. 

A study by Pabla et al. identified PKC*δ* as a critical regulator of cisplatin nephrotoxicity, which can be effectively targeted for renoprotection during chemotherapy. The data showed that during cisplatin nephrotoxicity, Src interacted with, phosphorylated, and activated PKC*δ* in mouse kidney lysates. After activation, PKC*δ* regulated MAPKs, but not p53, to induce renal cell apoptosis. Thus, inhibition of PKC*δ*, pharmacologically or genetically, attenuated kidney cell apoptosis and tissue damage, preserving renal function during cisplatin treatment. Conversely, inhibition of PKC*δ* enhanced cisplatin-induced cell death in multiple cancer cell lines and, remarkably, enhanced the chemotherapeutic effects of cisplatin in several xenograft and syngeneic mouse tumour models while protecting kidneys from nephrotoxicity. Together these results demonstrate a role of PKC*δ* in cisplatin nephrotoxicity and support targeting PKC*δ* as an effective strategy for renoprotection during cisplatin-based cancer therapy [133].

## 7. Nonsteroidal Anti-Inflammatory Drug- (NSAID-) Induced Kidney Injury

### 7.1. NSAIDS

The NSAID family includes several classes of drugs such as the carboxylic acids, for example, aspirin; acetic acids, for example, diclofenac; propionic acids, for example, ibuprofen and ketoprofen; and Cox-2 inhibitors, for example, celecoxib which are used worldwide as analgesics and antipyretics to combat pain, fever, and inflammation. They are especially effective for treating inflammatory diseases (e.g., arthritis) through nonspecific inhibition of cyclooxygenase (COX) enzymes which limits production of prostaglandins. Serious gastrointestinal side effects have been minimized with the advent of selective and specific COX-2 inhibitors and misoprostol. However, the newer NSAIDs continue to be nephrotoxic much like the conventional NSAIDs [134]. The spectrum of nephrotoxicity includes acute tubular necrosis, acute tubulointerstitial nephritis, glomerulonephritis, renal papillary necrosis, chronic renal failure, salt and water retention, hypertension, and hyperkalaemia [135, 136].

### 7.2. MAPK in NSAID Nephrotoxicity

Hou et al. investigated the molecular basis of the renal injury by evaluating the expression of the stress marker, haeme oxygenase-1 (HO-1), in celecoxib-stimulated glomerular mesangial cells [137]. Treatment with celecoxib resulted in concentration- and time-dependent increase of HO-1 expression. Conversely treatment with *N*-acetylcysteine, a free radical scavenger, strongly decreased HO-1 expression, suggesting the involvement of reactive oxygen species (ROS). Following treatment with various MAPK inhibitors, the study showed that only a specific JNK inhibitor attenuated celecoxib-induced HO-1 expression, and kinase assays demonstrated increased phosphorylation and activation of c-JNK following NSAID treatment [137]. The presence of a free radical scavenger reduced the stimulatory effect of celecoxib on stress kinase activities, suggesting an involvement of JNK in HO-1 expression. Treatment with a PI-3K specific inhibitor prevented the enhancement of HO-1 expression, which correlated with inhibition of the phosphorylation of the PDK-1 downstream substrate Akt/protein kinase B (PKB). The data presented in this study suggested that celecoxib-induced HO-1 expression in glomerular mesangial cells may be mediated by ROS via the JNK-PI-3K cascade [137].

## 8. Conclusion

The complex nature of critical illness often necessitates the use of multiple therapeutic agents, many of which may individually or in combination have the potential to cause kidney injury. Accordingly, the incidence of drug-induced nephrotoxicity is rapidly increasing [138]. Drugs known to cause nephrotoxicity have been shown to exert their toxic effects through one or more common pathogenic mechanisms [139]. It is important to appreciate that a single drug causing renal toxicity can involve multiple pathophysiological pathways and that predisposing factors are common to virtually all causative agents mediating kidney injury. Various studies, both *in vitro* and *in vivo*, have shown that the administration of certain drugs acts as the stimulus to trigger various MAPK cascades, thus mediating cellular responses to kidney injury [139]. Indeed, activation of these central pathways is evidenced in both acute and chronic kidney injury. Data from renal biopsies in humans have shown upregulation of MAPKs in a variety of renal conditions, suggesting involvement in human renal disease, and may provide a new target for intervention. Interventions aimed at providing renoprotection, such as ACE-inhibition or statin therapy, can reduce the renal MAPK expression, suggesting that increased renal MAPK expression is involved in the pathophysiology of kidney damage. The use of specific MAPK inhibitors has further elucidated this role. Animal data presented in this paper suggests that MAPK inhibition may be of use in acute inflammatory renal disorders, and in chronic conditions characterized by fibrosis. In order to explore the potential of MAPKs as a novel intervention strategy in kidney disease, it is important to establish the renal conditions that can specifically benefit from MAPK inhibition, since studies have shown that not all conditions can be improved through inhibition of the MAPK signalling cascade [24]. However, since the MAPK cascades have been implicated in numerous studies in the development of kidney damage and disease, continued research in this area will hopefully highlight novel therapies or mechanisms of prevention of kidney injury.
